# Biophysically Realistic Filament Bending Dynamics in Agent-Based Biological Simulation

**DOI:** 10.1371/journal.pone.0004748

**Published:** 2009-03-13

**Authors:** Jonathan B. Alberts

**Affiliations:** Center for Cell Dynamics, Department of Biology, University of Washington, Seattle Washington, United States of America; University of East Piedmont, Italy

## Abstract

An appealing tool for study of the complex biological behaviors that can emerge from networks of simple molecular interactions is an agent-based, computational simulation that explicitly tracks small-scale local interactions – following thousands to millions of states through time. For many critical cell processes (e.g. cytokinetic furrow specification, nuclear centration, cytokinesis), the flexible nature of cytoskeletal filaments is likely to be critical. Any computer model that hopes to explain the complex emergent behaviors in these processes therefore needs to encode filament flexibility in a realistic manner. Here I present a numerically convenient and biophysically realistic method for modeling cytoskeletal filament flexibility *in silico*. Each cytoskeletal filament is represented by a series of rigid segments linked end-to-end in series with a variable attachment point for the translational elastic element. This connection scheme allows an empirically tuning, for a wide range of segment sizes, viscosities, and time-steps, that endows any filament species with the experimentally observed (or theoretically expected) static force deflection, relaxation time-constant, and thermal writhing motions. I additionally employ a unique pair of elastic elements – one representing the axial and the other the bending rigidity– that formulate the restoring force in terms of single time-step constraint resolution. This method is highly local –adjacent rigid segments of a filament only interact with one another through constraint forces—and is thus well-suited to simulations in which arbitrary additional forces (e.g. those representing interactions of a filament with other bodies or cross-links / entanglements between filaments) may be present. Implementation in code is straightforward; Java source code is available at www.celldynamics.org.

## Introduction

Complex behaviors in cells often emerge from surprisingly simple sets of underlying molecular interactions. Understanding how such emergent behaviors arise from well-described biochemistry, geometry, and mechanics is a current focus in the field of computational and mathematical biology. It is only through such a rigorous formalization of our thinking about cellular systems that the major reductionist agenda in biology (disassembling systems into their fundamental molecular components) can be reconciled with the emergence of novel properties during the process of cellular self-organization, so that the system can be properly understood.

### Agent-based models

One useful tool for understanding emergence in cellular biology is agent-based computer simulation. In such modeling, the state (e.g. position and orientation in 3-dimensional space, biochemical activation or hydrolysis condition) of each primary component in a network of interactions is followed through time in a computer, typically using a large number of very small time-steps to integrate the governing system of differential equations. The calculated interactions of components can alter biochemical states, create complexes of components with new properties, deplete or enrich scalar concentration fields of soluble components, and generate forces that repel, attract, or deform. This tracking of spatial and biochemical detail can reveal dynamic behavior important in cell function. But both considerable computer power and many biological details (e.g., rate and equilibrium constants for all or most of the molecular interactions) are required for the informative use of such methods. Complex models of this type have been applied to actin-based motility [1], [2], spindle-pole positioning and oscillations [3], the role of motors in mitotic spindle formation [4]–[7], load sharing in Brownian ratchet mechanisms [8], and to understanding cytokinetic furrow specification [9], to select a few.

Detailed agent-based molecular mechanics models of cellular processes share some common challenges. For example, such simulations often track the states of a very large number of agents, and the modeler must determine and resolve collisions between these agents in an efficient way. In [1], we presented one efficient collision scheme (of many that are likely possible). To produce accurate *in silico* representations of biological features and increase the use of these methods, other techniques for overcoming computational hurdles will need to be developed, tested, and standardized. To this end, I present a method for representing cytoskeletal filaments *in silico*. An implementation in Java code (*SimFil*) that demonstrates features of this method and provides example representations for actin filaments, microtubules, and other biological filaments is available as a tutorial at www.celldynamics.org.

### Cytoskeletal filaments

There are three major classes of cytoskeletal filaments: microtubules, actin filaments, and intermediate filaments. None is a homogeneous, isotropic material, as each is formed from chains of discrete protein monomers [10]. At the typical level of description in molecular mechanics models, however, we can treat them as traditional engineering elements [11]; that is, they are considered to be Euler elastica whose mechanical behavior under axial and bending stress is described by an elastic modulus (Young's modulus) and a bending rigidity, respectively. The elastic moduli (*E*) of actin filaments and microtubules are large, similar to plastic [11], and individual cytoskeletal elements typically experience forces in the piconewton range (e.g. a motor protein will stall at a few pN). Under such force magnitudes, these filaments are essentially inextensible; thus, a molecular mechanics model can typically regard filament arc length as constant under force (For example, a 1 pN axial force will stretch a 1 µm long actin filament by only 0.02 nM, about one hundredth of a monomer radius (stiffness = *EA/L*, where *E* = 2.3 GPa, cross-sectional area *A* = 20 nm^2^, and length *L* = 1 µm)).

In contrast, filament deformations caused by bending will very often be critical to a molecular mechanics description. Although these filaments have large elastic moduli, they have small cross-sectional areas, and it is the product of the elastic modulus and the second area moment of inertia (*I*) that defines the bending rigidity, *EI*. Thus, thermal forces alone will deform cytoskeletal filaments from their unstrained straight configuration. Unlike the tiny axial change calculated above, the same 1 pN force applied transversely to a four-fold shorter 0.25 µm actin filament, with a cantilever support at one end (as in Fig. S1), will cause a considerable deflection of about 31 monomer radii. The biophysical properties of cellular filaments are discussed further in Text S1.

### The Proposed Method

I approximate flexible cytoskeletal filaments as a chain of rigid segments, each linked end-to-end by one translational and one torsional elastic element. The translational spring enforces the constraint that the endpoints of adjacent segments should be coincident, while a weighted sum of the torques from the translational spring and the torsional spring are used to approximate the bending rigidity of the filament (henceforce the endpoint and angular alignment constraints, respectively). It is the manner in which these segments are connected that allows the tuning to biophysically realistic behaviors.

The key goal is to mimic the biophysical behaviors of cellular filaments through a system of equations that is easy and fast to solve numerically, chiefly by not being stiff (i.e. prone to numerical instabilities). This goal is achieved by using unique elastic elements in which the restoring force is formulated in terms of single time-step constraint resolution. This formulation is only possible because of the special nature of the differential equations at low Reynolds number. That is, by neglecting inertial terms we are left with a set of first-order equations in which the history of motion is unimportant; bodies respond instantaneously (on the temporal scale of our numerical integration) to incident forces.

## Methods

### Pairs Derivation

The Pairwise Agent Interaction with Rational Superposition (PAIRS) method is so called because each force interaction is considered in isolation. The actual force state on each agent is, by the principle of superposition, taken as the sum of all the pairwise forces. The pairwise forces are formulated in terms of the force (or torque) required to resolve a system contraint in a single simulation time-step. To introduce this method, I first apply it to the fast (relative to the time-step) resolution of collisions between rigid bodies. I then show how the same technique can capture dynamics that are slow relative to the time-step by modeling a viscous spring. Finally, I derive the PAIRS force and torque for a segmented filament.

#### Two colliding spheres: dynamics fast relative to the time-step

The genesis of the PAIRS approach in [1] was the need for a non-stiff method to keep rigid bodies apart, i.e. resolution of collisions. We assume here that collisions are resolved faster than the simulation time-step. It is appropriate to neglect inertial terms for small cellular bodies, and by doing so we can calculate and apply a force that will perfectly resolve a collision (absent Brownian motion and other external forces) in a single time-step.

**Figure 1:**
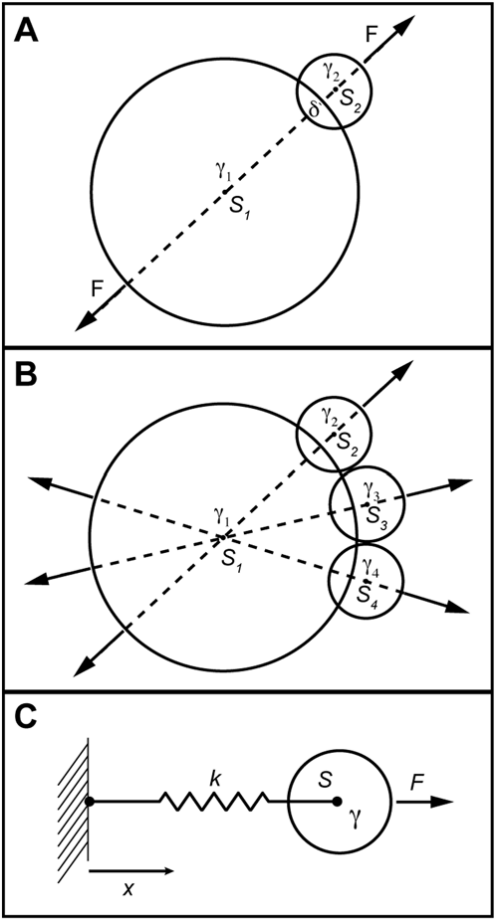
Colliding spheres demonstrate PAIRS method stability and failures. In A, calculation of the force *F* required to separate two spheres in an actual pairwise interaction (i.e. no other forces besides *F* are present) yields a stable collision resolution that can be accomplished in a single time-step, if desired. In B, the sum of the forces on 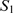, from collisions with the three other spheres, can lead to an overshoot, and non-convergent oscillations for some geometries. To avoid this failure, the PAIRS forces should be applied fractionally, such that collisions are resolved over multiple time-steps. C shows sphere 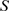 with drag 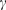 attached to a fixed wall by spring 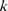. For an applied external force 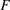 the equilibrium position is 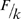 (assuming the spring is unstrained at x = 0) and the system should relax from that equilibrium, once the force is removed, with time-constant 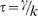. Modeling this simplest dynamic system by the PAIRS method involved replacing the spring force with a force calculated to return the system to its relaxed position in a single time-step. That force is then modulated by a PAIRS coefficient to allow tuning of the system to match physical properties of the system, such as expected deflection and relaxation time-constant.

Consider two colliding spheres 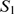 and 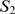 in Fig. 1A, with viscous drag coefficients 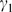 and 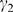. The spheres overlap by 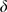 at time 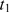 – we will determine the force that will just separate the spheres at 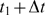.

First, write the force balance for 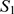, neglecting inertial terms…
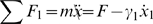
(1)If we use 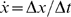 and solve for 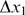 then
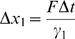
(2)We want to solve for the force ***F*** such that 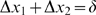, i.e. the force ***F*** will just push the spheres apart in a single time-step. Solving for ***F*** we find
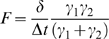
(3)


The *PAIRS* method superposes forces from each pairwise agent interaction. If no other forces than ***F*** are acting on the two spheres in Fig. 1A, then this method of collision resolution is stable at any time-step. No stability analysis is necessary to show this: since ***F*** is calculated to just separate the spheres it is not possible to overshoot the equilibrium position.

If multiple bodies are colliding with each other (Fig. 1B), as is often the case in any biological simulation, application of the entire force in equation 3 can lead to an overshoot and/or oscillation about the desired equilibrium. Consider the forces from spheres 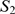, 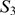, and 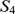 on 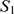 in Fig. 1B. By the *PAIRS* method, each interaction is considered in isolation and the sum of the forces on 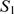 will cause that sphere to move farther than necessary to resolve the collisions with the smaller spheres. The degree to which 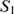 over-shoots the correct position depends on the magnitude of 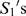 drag relative to the drag of the smaller spheres; in the limit where 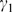 is very much larger than any other drag coefficient, the method actually works perfectly again. This potential failure of the *PAIRS* method results from the coincidental alignment of forces from multiple pairwise interactions. The degree of this failure depends on the geometry of the interactions and the properties (e.g. drag coefficients) of the interacting agents.

This problem with “interaction density” is general to explicit numerical methods –it is not unique to the PAIRS approach. There are a number of ways to avoid such overshoots and oscillations. Given knowledge of a maximum expected interaction density a constant and conservative fraction of the force in equation 3 could be applied over any time-step. Alternatively, one might adopt a scheme to actively determine the interaction density at each time-step and attenuate the force in equation 3 accordingly, i.e. an adaptive PAIRS coefficient scheme. Adaptive time-step schemes, useful with other explicit numerical methods, do not help here since the PAIRS force is explicitly time-step dependent.

#### A viscous spring: dynamics slow relative to the time-step

Next consider the simple viscous-spring system shown in Fig. 1C. I show how the PAIRS solution, once tuned to have the proper static deflection and time-constant, is identical to the conventional approach for this trivial example. By the conventional approach we would derive an equation of motion (neglecting inertial terms)
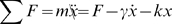
(4)The equilibrium displacement of this system under a constant force 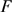 is found by setting 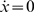, leading to 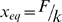. By assuming a solution of the form 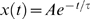 once 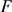 is removed, we find the system time-constant as 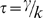. This equilibrium displacement and time-constant will be our targets for tuning the PAIRS coefficient.

By the PAIRS method we first consider the natural system (i.e. without external force 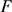, neglecting inertial terms), and replace the spring force by a force 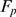.
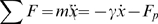
(5)We then calculate a value for 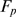 such that the system, perturbed by 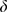, moves to its relaxed position in a single time-step
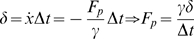
(6)At this point we introduce a PAIRS coefficient, 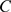, to tune the system response to match our deflection and time-constant targets, i.e. 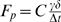. Instead of a particular perturbation, replace 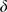 with any displacement 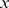 and substitute this value for 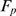 into equation 5 to obtain the discrete equation of motion
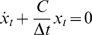
(7)If we make the forward Euler substitution of 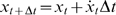 then the numerical solution for 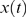 is
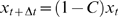
(8)This solution for 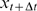 is stable provided that 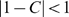; the *PAIRS* coefficient ***C*** is thus restricted to be less than 1, a general result for PAIRS method representations of more complex dynamics, such as the hydrodynamic flexible beam.

There is no time-step dependence in equation 8, which might be initially worrisome as the numerical solution does not seem to converge, i.e. how can this numerical scheme approach the exact solution as the time-step goes to zero if there is no explicit time-step dependence? Resolution is found in the nature of the PAIRS coefficient 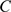, since that coefficient is actually a function of time-step, i.e. 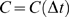. If we impose either the deflection or time-constant constraints (by reintroducing an external force into equation 7 and requiring that the solution for 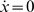 match the expected deflection 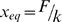, or by substituting 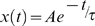 into equation 7 and requiring that 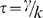) we find that
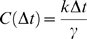
(9)Thus, substitution into equation 8 of the proper PAIRS coefficient (for the correct deflection and time-constant) gives a numerical master equation identical to the conventional approach, and thereby identically convergent.

#### Derivation of Pairwise Agent Interactions with Rational Superposition (PAIRS) force and torque for connected filament segments

**Figure 2:**
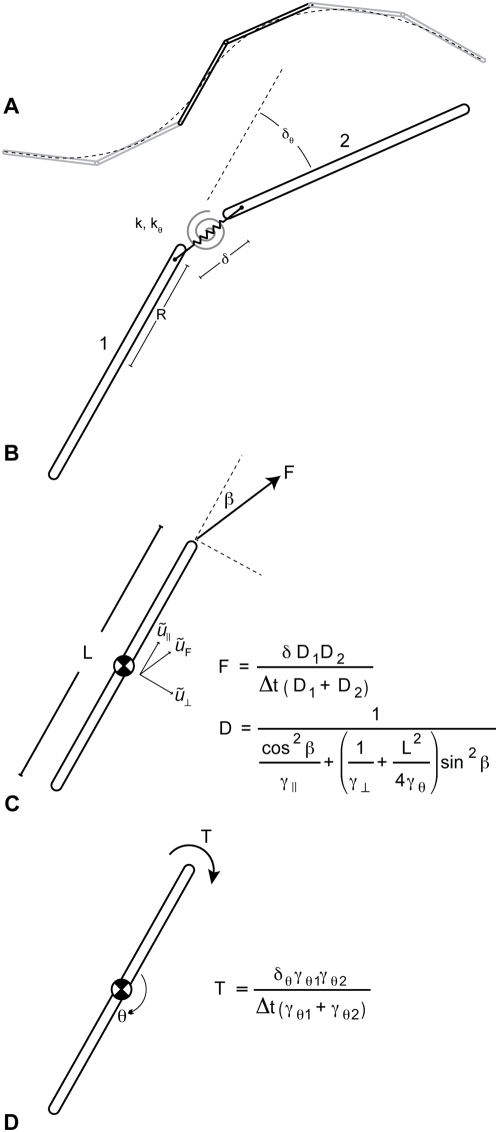
A lumped-parameter representation of an Euler elastica (dotted line in A) by a chain of straight segments. To avoid the computationally expensive algebra that enforces exact alignment of the segment endpoints (as in A), we let segment ends drift apart slightly at each time-step (as in B), and install springs that act to correct that drift and pull separated endpoints back together again. The translational spring 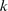, unstrained when 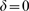, applies an axial force to bring endpoints together. The torsional spring 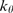, unstrained when 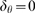, applies a torque to co-align adjacent segments. The axial force acts through a point a distance 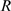 from the segment centroid. Unique elastic elements generate forces that allow the model to resolve constraints in a single time-step: the Pairwise Agent Interaction with Rational Superposition (PAIRS) method. (C) Two rigid segments, joined by a translational and a rotational elastic element, form the basic unit in computational simulation of a continuously flexible filament. (C–D) By the PAIRS method, the spring coefficients for the translational and rotational elastic elements are 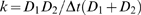 and 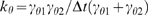, respectively, where 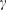 are the viscous drag coefficients in the different coordinate directions. The force and torque both take the form of 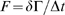 where 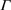 is the equivalent drag for two dissipative elements in series. See Methods for the derivation and discussion of the form of these results.

Consider the rigid segments that are part of a segmented representation of a continuously flexible filament as in Fig. 2A. I will now derive the PAIRS force and torque that will align segment endpoints and segment orientations, respectively, in a single time-step. Then I will introduce PAIRS coefficients, as with the viscous spring, that allow the tuning of filament behavior to match the biophysical targets of deflection and relaxation time-constant.

#### PAIRS axial force

The segments labeled 1 and 2 are pinned together at their endpoints, but we soften this hard constraint by introducing springs that work to keep segment endpoints coincident. We will approximate the value of force 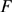 that will work to satisfy this pinned constraint by moving the endpoints back together in a single time-step. For a cytoskeletal filament, this pinned constraint enforces a constant filament arc length; since cytoskeletal filaments are very stiff longitudinally their arc lengths should not change appreciably under cellular force magnitudes.

The derivation sums the displacements of each pinned endpoint, along the line of action 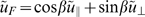, due to force 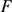. We then find the value of 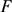 such that this sum of displacements is equal to the current misalignment distance 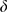. Begin by expressing the force 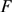 in body-fixed components for segment 1…
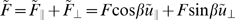
(10)where 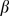 is the angle between the filament and the line of action of the force. We have omitted a subscript 1 from 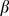 and the drag terms 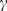 below for simplicity in the notation. Since the summation of forces on each rigid element has a viscous term 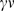, we use 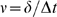 and express the displacement in any coordinate direction as 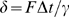. Consider also the rotational displacement of endpoint 1 from the torque applied by 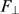, which for small time-steps is wholly in the 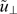 direction. This torque has magnitude 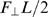 and for small angles the translation in the 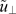 direction from an angular rotation 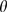 is 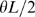. The total displacement of endpoint 1 in body-fixed coordinates is then
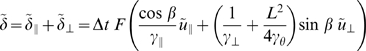
(11)where 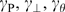 are the viscous drags for axial, transverse, and rotational motions, as shown in Fig. 2C and D. The dot product of this displacement vector with the vector 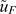 gives the displacement in that direction for endpoint 1
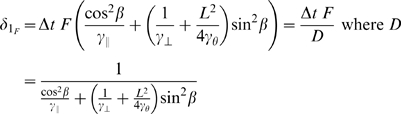
(12)An identical derivation holds for endpoint 2, and we set the sum of these to the misalignment distance
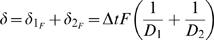
(13)Solving finally for 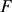 we have
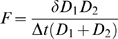
(14)


#### PAIRS torque

Here we calculate the torque *T* that will, in a single time-step 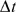, align segments 1 and 2 that are misaligned by angle 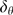. In contrast to the pinned constraint, alignment of adjacent segments represents the much softer spring of a beam in bending. While we are deriving the torque that will align segments in a single time-step, we will in practice use a PAIRS coefficient to accomplish this relaxation to an unstrained state (i.e. a straight filament) in many time-steps, as defined by the system time-constant.

The angular rotation of segment 1 due the torque *T* is just
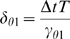
(15)and likewise for segment 2. Setting the sum of these rotations to the misalignment angle, we can solve for *T*

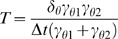
(16)


#### Equivalent spring constants

The results from equations 14 and 16 can alternately be written in terms of special spring constants, which depend on time-step, filament orientation, and drag coefficients:
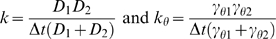
(17)These derivations consider the contribution to translational or angular displacement from each of the two linked segments. In the torque calculation, this contribution is only a function of the rotational drag of each segment (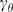). In the force calculation the displacement contribution depends on both drag coefficients and the particular orientation of the segment.

#### Equivalent drags

The final form for both force and torque is perhaps best understood as being in the form
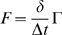
(18)where 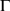 is the equivalent drag of the two elements. The equivalent drag for two viscous elements in series (analogous to the equivalent resistance of two electrical resistors in parallel) is given by the reciprocal of the sum of reciprocals, i.e.
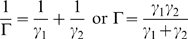
(19)This form is apparent in both the derived force and torque expressions, though the *D* terms in the force expressions mask some underlying details.

### Pairs Coefficient Tuning

Equations 14 and 16 reveal the force and torque necessary to align endpoints and orientations, respectively, in a single time-step. By introducing and tuning three PAIRS coefficients and two Brownian force related coefficients, this flexible filament representation can capture the biophysical essence of actin (and actin-like) filaments, microtubules, and other polymers


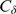
 **– PAIRS force attenuation:** In practice, the PAIRS force in equation 14 will be attenuated through the coefficient 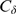. Instead of attempting to align adjacent filament segment endpoints in a single time-step, this coefficient “softens” that constraint. For a filament composed of identical rigid segments, this coefficient should, theoretically, be less than 0.5. This will maintain an over-damped response if the PAIRS forces from each of the two adjacent and connected segments are aligned.


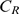
 **– The PAIRS force torque arm:** As indicated in Fig. 2B, the PAIRS force is applied at a tunable distance R from the segment centroid. The tuning coefficient 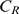 is the fractional distance from the centroid at which the PAIRS force is applied, i.e. if 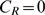 then the force is applied at the centroid and if 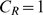 then the force is applied at the segment endpoint. In practice, 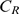 can range higher than 1, dependent upon the value of 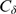. The degree of freedom afforded by 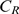 is critical – it allows us to mimic biophysical behaviors of different filaments at different segment sizes, time-steps, and viscosities.


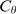
 **– PAIRS torque attenuation:** The PAIRS torque in equation 16 is, in practice, attenuated through the coefficient 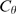. As with the PAIRS force, the theoretical upper limit for 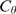 is 0.5, i.e. for identical linked segments any value less than this maintains an over-damped response in case the PAIRS torques from each adjoining segment are aligned.


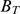
 **– Translational Brownian forcing:** This coefficient scales the translational Brownian motion of a rigid segment that is part of a larger filament. The reference force is the force that would be applied to identical free segment, i.e. 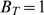 specifies translational forcing identical to that for a free segment.


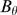
 **– Rotational Brownian forcing:** This coefficient scales the rotational Brownian motion of a rigid segment that is part of a larger filament. The reference torque is the torque that would be applied to identical free segment, i.e. 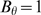 specifies rotational forcing identical to that for a free segment.

#### The Tuning Procedure

In equation 9 we found a simple relationship between the PAIRS coefficient introduced in that analysis and the spring and damping constants for a viscous spring. For the more complex dynamical representation of the hydrodynamic beam equation –the focus of this manuscript– we cannot derive analytical expressions for 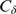, 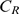, and 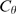; those tuning coefficients are empirically chosen to compensate for the coarse spatial discretization of a filament (choice of segment size) and for the lumping of continuous elastic properties into discrete elements.

#### Tuning for deflection and relaxation time-constant

To tune *in silico* filaments to the correct static deflection and relaxation time-constant, adjust 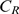 and 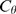. 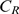 and 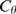 multiply the two independent constraint torques and independently affect both deflection and time-constant –they must be empirically tuned in concert to match both behaviors. I use the following procedure with the *SimFil* code to find appropriate PAIRS tunings for 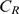 and 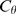.

1. Set 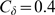, an arbitrary value close to, but less than 0.5.
2. Set 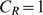, an arbitrary starting guess
3. Adjust 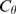 till deflection 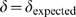 (for a point load at the midpoint of a simply-supported filament as in Fig. S1).
4. Relax the supports to measure the relaxation time-constant, 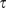.
5. Decrease 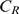 if 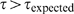, and vice versa.
6. Repeat steps 3 through 5 till 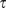 and 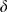 are within the desired tolerance of their expected values. If either 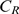 or 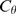 is outside of its stable range, a different filament discretization and/or time-step must be used.

#### Tuning for thermal motions

Since all filament motions depend on the PAIRS coefficients 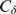, 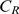, and 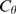, tuning for the appropriate degree of thermal writhing (i.e. time-averaged angular correlations between segments of the same filament) must be done after the tuning for deflection and relaxation time-constant. I use two coefficients, 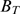 and 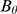, to independently adjust the magnitude of a random force and torque, respectively, that are used to approximate the Brownian forces on each rigid segment. The reference force and torque, i.e. when 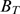 and 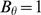, is calculated to simulate the Brownian motion of a free (unlinked) rigid segment. I use the following procedure with the *SimFil* code to find appropriate tunings for 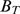 and 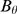:

1. Start with 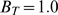 and 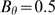 –final values are usually close to these.
2. Record the time-averaged angular correlations (i.e. the dot product of segment unit vectors) between the first and all subsequent segments over a simulation period that is much larger than the filament relaxation time.
3. Compare the result with the expected curve 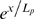.
4. If the slope of the interior segments is too shallow, increase 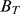, and vice versa. If the slope of the end segments is too shallow, increase 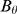, and vice versa.
5. Repeat steps 2 through 4 until the simulated and expected angular correlations are within the desired tolerance.

I have empirically found that this method works best if 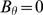 for internal segments, and this is how the application of Brownian forces in currently implemented in *SimFil*. This *ad hoc* technique for matching thermal writhing is limited to filaments comprising a small number (<20) of rigid segments, which should be considered when choosing a segment size (see Discussion). This limitation is an artifact of the simple scheme I use in applying Brownian forces and toques; a different / more sophisticated approach should be developed, e.g. applying correlated, instead of random, Brownian forces to adjacent segments.

#### Solution Procedure

**Figure 3:**
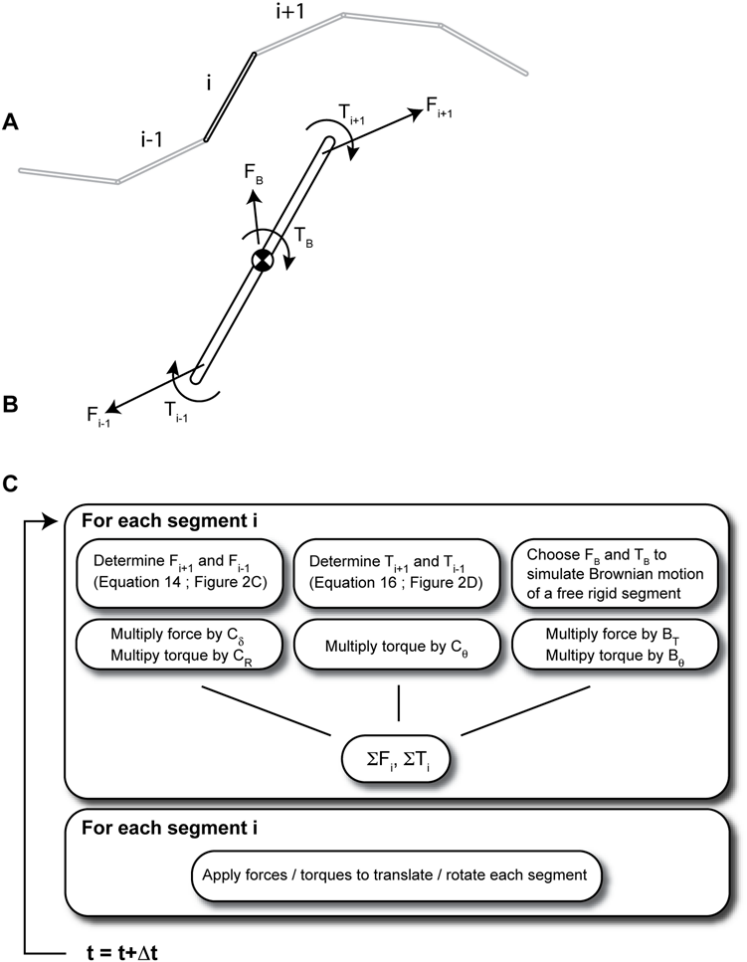
Synopsis of the solution method. The constraints between rigid segments in an *in silico* representation of a continuous filament (A) are applied through the forces shown in the free-body diagram of a single segment (B). The random force and torque, 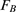 and 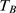, generate an appropriate Brownian motion. The flow chart in C summarizes the solution steps: (1) determine all forces for the set of segment positions at time 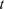, (2) apply the PAIRS coefficients, (3) move each segment to time 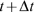 through application of Newton's second law, neglecting inertial terms.

Fig. 3 graphically summarizes the solution procedure for a single flexible filament. Newton's laws are applied –after a summation of PAIRS and Brownian forces / torques– to move each filament segment forward in time. External forces from interactions with other filaments, motor proteins, cross-linkers, etc could be considered as well.

## Results

### An example tuning

**Figure 4:**
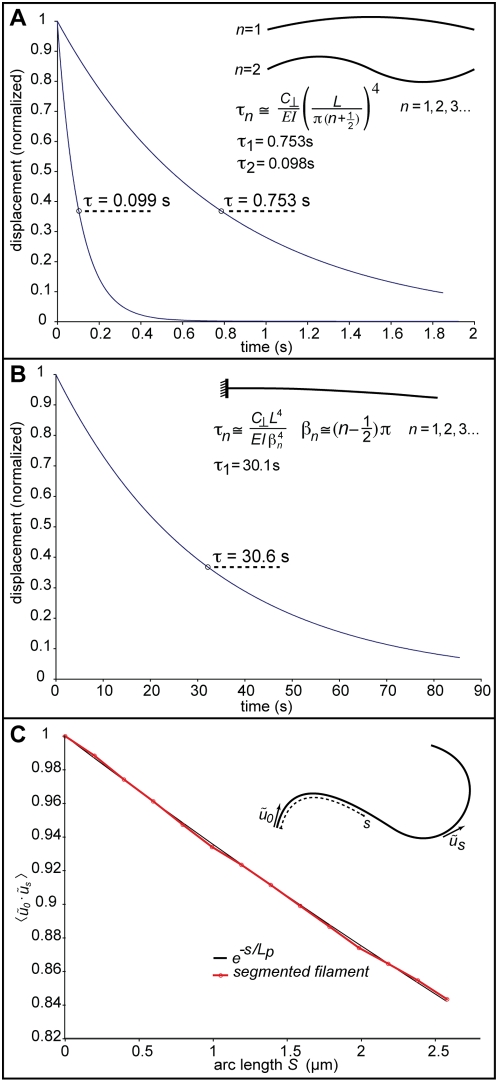
The biophysical targets, and results from one implementation with ParM filaments. (A) The expected and *in silico* 1^st^ and 2^nd^ mode time-constants for a free 3.2 µm (13 segments of 100 monomers *in silico*) ParM filament in a viscosity 100 times that of water, located 0.5 microns from a plane surface. The expected values are from solutions to the hydrodynamic beam equation. The tuning coefficients 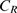 and 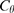 are adjusted such that both the static deflection for a simply-supported beam (see Fig. S1) and the 1^st^ mode time-constant (when the applied force and supports are simultaneously removed) match expectations. Unlike the 1^st^ mode free time-constant, the 2^nd^ mode free and the 1^st^ mode cantilevered time-constants (B) are not involved in the tuning, yet the *in silico* representation faithfully reproduces those responses. In these tests an external force and boundary conditions are applied to a filament. After equilibrium is achieved the force and boundary conditions are removed and the relaxation time to displacement 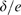 is measured. (C) Independent of the deflection and time-constant tuning, the magnitude of the rotational and translational Brownian forces can be adjusted to achieve expected angular correlations between rigid segments, using a persistence length of 15 µm for ParM filaments.

ParM is a bacterial protein that forms actin-like filaments [12]. Fig. 4 shows the results of this tuning for a 3.2 µm long ParM filament (*L_p_* = 15 µm) comprising 13 rigid 100-monomer segments, and 0.5 microns from the nearest surface. I adjust the tuning coefficients 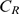 and 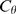 to match, simultaneously, the expected static deflection for a simply-supported beam (Fig. S1) and a free time-constant of 0.753 seconds for relaxation from the 1^st^ bending mode. The coefficient 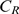 is constrained to the range 0 to 1.5, and a value of 1 is preferred. Therefore, the tuning procedure begins with 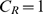 and 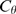 is adjusted (within a range from 0 to 0.5) to get the correct deflection. If the resulting time-constant is too slow, decrease 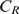, and vice versa. To demonstrate that this method accurately represents the bending elasticity of filaments, rather than simply matching the tuning criteria in a narrow region, Fig. 4A and 4B show that, with the same values of 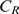 and 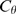, we also reproduce the 2^nd^ mode free and 1^st^ mode cantilevered time-constants. Fig. 4C shows the close match that is possible between simulated and expected angular correlations along a 3.2 µm ParM filament.

### Choice of filament discretization

Several competing concerns must be considered together when choosing a rigid segment size. Large segments will reduce the number of independent bodies in the simulation and lead to faster run times. Smaller segments better capture tight bends and better match expected deflections and time-constants for shorter filament lengths. Additionally, constraints from the tuning method (i.e. inability to achieve proper deflection and time-constant with the practical range of 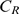 and 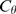 values) may force an increase or decrease of segment length, if a particular time-step is desired. A limitation in matching the expected angular correlations from thermal writhing, discussed in Methods, might put a lower limit on segment size by requiring the longest filament to comprise no more than 20 segments, if that biophysical behavior is deemed critical.

As an example of balancing these competing criteria, consider my choice of a 100-monomer (at 2.45 nm per monomer) rigid segment size in simulating ParM mediated segregation of plasmids in *E. coli* with longest axis of 3 µm. At ∼0.25 microns per rigid segment, the longest filaments will comprise no more than 13 segments and will exhibit appropriate thermal writhing. A 0.25 µm segment of the ParM filament is by no means rigid under piconewton forces (see calculation in the Introduction), but I assume that deflections of filaments shorter that this are not important in the overall emergent behavior –a decision that I have substantiated by a few computationally more expensive simulations with a smaller segment length. The entire filamentous ParM population will comprise just over one hundred segments at the steady-state ParM concentration, allowing very rapid collision detection and solution of the associated equations of motion. (Twenty minutes of simulated time, a typical division period for *E. coli* in the associated experiments, can be accomplished in just a few hours of computer time).

### Dependencies and errors

It is important to note that this tuning procedure assumes a particular time-step, segment size, filament length and viscosity; each of these values can greatly affect the 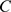 and 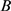 tuning coefficients. This does not mean, however, that a simulation is limited to constant time-step, segment size, or viscous environment. Variable time-step numerical integration schemes, changing viscous environments (e.g. a simulation in which proximity of a filament to a surface modulates viscous drag), and different segment size filaments (e.g. a simulation in which spatial location dictates filament tessellation) can be accomplished with look up tables or functions that are fit to the tuning coefficients across the range of variability.

**Figure 5:**
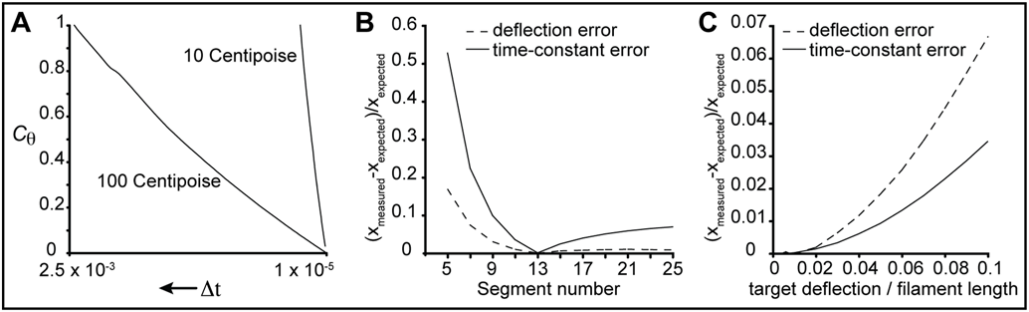
Dependencies of PAIRS coefficients and method on time-step, filament length, and force. The reference filament for all the results presented here is a 3.2 µm ParM filament represented by 13 rigid segments, each 100 monomers long (the example tuning from Methods and the *SimFil* default parameter set). A demonstrates, numerically, the near linear dependence of 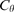 on 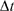 at two different viscosities (10× and 100× water). A variable time-step method could thus use look-up tables or functions to set 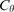, i.e. for a given segment size and viscosity we could establish 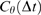. B shows the error in deflection (for a simply-supported filament) and first mode free time-constant as a function of filament length. The PAIRS coefficient tuning was for a filament of 13 rigid segments. The errors –reported as the difference between actual and expected values divided by the expected value– are reasonable for long filaments, though errors grow large for filaments with very few segments, as might be expected. The absolute errors are reported here but filaments shorter than the tuning point are stiffer and slower to relax than expected, and vice versa for longer filaments. C shows the error in deflection (for a simply-supported filament) and first mode free time-constant as a function of the magnitude of the test force. The test force is calculated as the theoretical force required to deflect a simply-supported filament (see Fig. S1) to a target deflection –the abscissa is thus reported as target deflection divided by filament length. Over an order of magnitude change in force in either direction from the tuning point (tuning point = 0.01, i.e. a 1% deflection) the error remains small. Errors increase to a few percent as the test force gets very large (0.1, i.e. a 10% deflection). Filaments are stiffer and slower to relax than expected at large test forces.

Fig. 5 explores the PAIRS coefficient dependence on time-step and the method accuracy as a function of filament length and external force. For filaments both shorter and longer than the tuning length (from 1 to 6 µm), the 1^st^ mode free time-constant varies by only a few percent from the expected value (Fig. 5B). However, the error in both deflection and time-constant grows large for filaments comprising a very small number of rigid segments, as might be expected (Fig. 5B). The PAIRS method develops only a small error for forces that deflect a simply-supported beam 0.1 to 10% of its length (Fig. 5C).

## Discussion

A modeler will not typically be sure, a priori, which biophysical realisms are appropriate in a model of any particular biological system. This is because the emergent property under study may depend critically on one set, but not on other sets, of component interactions. For instance, a modeler might find that the particular behaviors of a motor protein were unimportant, so long as the motor's processivity and stall force were of the right magnitude. Alternatively the specific way in which a cortically attached motor protein regulates filament length could be essential to an emergent behavior [9]. The flexibility of cytoskeletal filaments, however, is likely a necessary and critical component for many cell processes. Techniques for realistically modeling this flexibility in agent-based simulations therefore need to be tested and standardized. One such technique forms the basis for this communication.

The method I propose for capturing the realistic biophysical behaviors of cytoskeletal filaments in agent-based computer simulations can match three critical properties of biological filaments: their deflection, relaxation time-constant, and thermal writhing (Fig. 4). These realistic filament properties emerge from an entirely local (pairwise) consideration of forces, making this method highly suitable for biological simulations with complex interactions, e.g. local modulation of filament stiffness by protein decoration, cross-linking of filaments, etc. This tuning to match biophysical properties could be achieved with any type of elastic elements, if segments are connected as in Fig. 2B – the variable attachment of the translation spring adds the critical degree of freedom. Such a system of rigid segments connected by simple springs could be solved by any number of explicit or implicit numerical schemes. To confront and evaluate numerical instability when using the forward Euler method (justified in Text S2), I additionally propose a novel pair of elastic elements to link a series of rigid segments that model a continuous filament.

With this method, a modeler balances the competing criteria of computational speed and coarseness of filament representation (plus several modeling subtleties) to determine an appropriate filament discretization (i.e., its number of rigid segments). For a particular chosen discretization, time-step, and fluid viscosity, a unique pair of tuning coefficients, 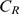 and 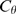 (see Methods), can be found that grant the *in silico* filament representation the proper deflection and relaxation time-constant. By the PAIRS method, analysis of numerical stability is immediate at any time-step, segment size, or viscosity: invariant to these properties, the tuning coefficient 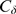 must range from 0 to 0.5, 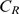 must range from 0 to 1.0 (or slightly larger), and 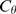 must range from 0 to 0.5 for stability. Two additional tuning coefficients, 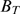 and 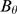, adjust the magnitude of the simulated Brownian force and torque, respectively, to achieve the expected degree of thermal writhing.

In practice, PAIRS forces are straightforward to implement (see Text S3 for pseudo-code example, or the *SimFil* source code at www.celldynamics.org), and they have numerical advantages beyond the aforementioned stability. Because each pair of linked segments is considered in isolation from other forces and links, the method scales well to any discretization of filaments (the rigid segment size in Fig. 2). This is not true in an exact matrix solution for each filament, where an increasingly large matrix must be inverted as the segment size is decreased. It is also trivial to alter the properties of particular sections of a filament by adjusting the forces for a subset of segments. A model that includes proteins that side-bind to filaments and modulate their mechanical properties (e.g. tropomyosin or cofilin on actin filaments) might well want to include this level of realism.

This way of representing filaments, in which the “agents” in the model are rigid subsections of a filament, deserves further comment. Any actual cellular process might involve many different cellular bodies, which interact with each other biochemically and/or mechanically. My approach is to break the large system of interacting components, which can have complicated and changing connection topologies, into a larger group of independent agents whose connections to each other, whether persistent or transient, are mediated by force. By considering filaments and other spatially dispersed cellular bodies as a large number of rigid agents, I handle all mechanistic interactions pairwise and locally. The force state on any agent with multiple pairwise interactions is determined by the principle of superposition, i.e. as the sum of the pairwise interaction forces. At each time-step, the local elastic forces naturally work to relax the entire interconnected system of agents to a lower energy state, consistent with the Principle of Minimum Total Potential Energy. A philosophically similar, but mathematically very different, methodology for implementing agent-based modeling of cellular processes is described in [13].

### Why does this tuning method work?

The two constraint torques, 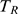 and 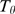, work to align the connected segments in different ways and with different time-scales. One of these torques, 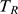, arises from enforcement of the endpoint constraint (equation 14), while the other is the PAIRS torque, 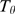, from equation 16. The magnitude of 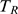 is proportional to endpoint, not angular, misalignment; typically 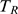 –which is the torque associated with the springs pulling segment endpoints together –slowly but incompletely aligns segments. By contrast, the PAIRS calculation of 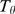 assumes the alignment of segments in a single time-step. The magnitude of this torque is reduced for numerical stability (i.e. 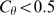), but the torque nevertheless provides relatively rapid co-alignment of the segments. The tuning coefficients 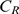 and 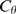 weight the alignment contributions from 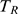 and 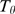, respectively, allowing any intermediate time-scale to be chosen. At some ratio 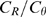 the system will have a time-constant that matches the one expected for the particular type of filament; but only one set of values 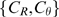 that satisfies this ratio will, in addition, deflect appropriately under external forces.

### Mathematical Justification and Limitations

The PAIRS method is best understood as a reformulation of elastic forces in terms of the “maximum stable restoring force” –the force required to move a dynamic system to its relaxed state in a single time-step. The trivial application of the PAIRS method to a viscous spring (equations 4–9) makes this interpretation clear. In that example, the Hookean spring force is replaced by a force calculated to move the system to its relaxed position in a single time-step. This force is then modulated by a PAIRS coefficient, chosen so that the static deflection and time-constant are as expected. For this simple system we can find an analytical expression for the PAIRS coefficient (equation 9), which reveals that we simply recover the same master numerical equation as by a conventional approach. The PAIRS method is thus identically convergent as a conventional explicit Euler approach.

The *PAIRS* formulation can consistently represent dynamics that are both fast and slow relative to the numerical time-step. In the case of fast dynamics (relative to the time-step), such as the longitudinal extension of biological filaments or the resolution of collisions, the *PAIRS* method is essentially enforcing a rigid constraint (e.g. no filament elongation, no impingement of rigid bodies) in a pairwise manner. By other solution methods these constraints might be formulated in terms of Lagrange multipliers, and would involve the simultaneous (as opposed to pairwise) solution of an entire system of equations. The enforcement of fast-dynamics constraints by the PAIRS method is invariant to time-step, viscosity, spatial discretization, etc, i.e. we can change these properties and the fast dynamics in a simulation will continue to be resolved as fast as is stably possible.

In the case of slow dynamics (relative to the time-step), the *PAIRS* method can capture, through a time-step dependent tuning of *PAIRS* coefficients, whichever biophysical properties the user deems most important. I have demonstrated such tunings for the case of biological filaments, choosing deflection and relaxation time-constant as the critical targets. By the PAIRS formulation, the tuning coefficient 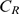 is dependent only on the spatial discretization of the filaments (i.e. on the choice of rigid segment size). Additionally, I can numerically show that the tuning coefficient 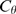 is linearly proportional to choice of time step (Fig. 5A) and inversely proportional to viscosity (data not shown).

The beam equation and hydrodynamic beam equation (for beams immersed in viscous fluid) are second-order ordinary and fourth-order partial differential equations, respectively, describing transverse displacements as a function of position along the beam as a function of beam properties (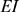 and 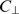) and applied forces (e.g. due to collisions, attached linkers, or motors) and applied moments. The *in silico* representation I propose –in order to approximate both this proper analytical description and the real filament behavior– considers only interactions between rigid bodies, elastically linked in series to form a beam, one segment pair at a time (Fig. 2). This representation is a form of lumped-parameter model; commonly in engineering and physics, the continuous axial and bending compliance of a beam are “lumped” into equivalent compliances at discrete locations along the beam. More broadly, I justify this representation as being conceptually identical to finite element modeling (i.e. a body is tessellated into a meshwork of elements with prescribed local elasticity, Poisson ratio, etc), a well-accepted practice for studying dynamics in bodies of complex shape and/or composition.

As is typical for finite element models, filaments may be discretized with arbitrary fineness. For example, given sufficient computational power the rigid elements in a representation of an actin filament could be any length, down to actin monomer-sized. It is important to note that the added complexity of viscous forces, considered by the hydrodynamic beam equation, are explicitly applied to each rigid element in our *in silico* representation.

### Applications

**Figure 6:**
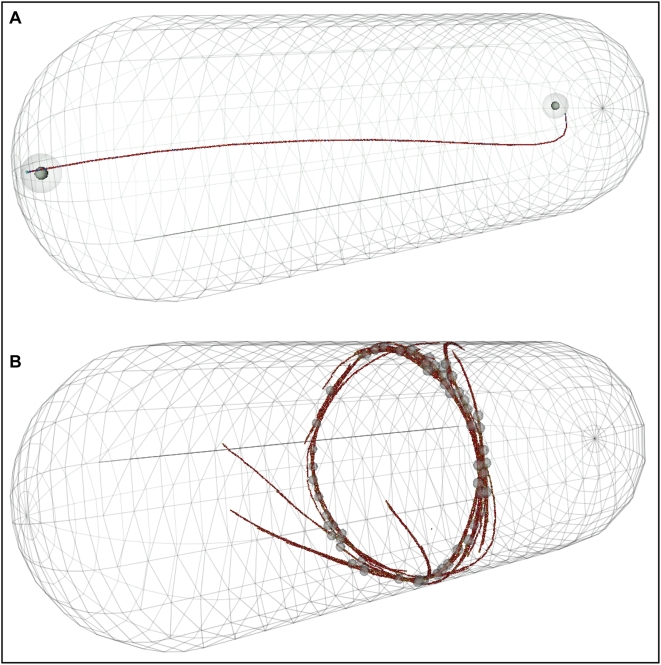
Applications of flexible filaments *in silico*. In A two gene-carrying plasmids in *E. coli* are segregated by a single ParM filament. Appropriate flexibility of this filament may be important in exploring the details of this primitive mitotic process. A large number of free (non-plasmid bound) ParM filaments, whose dynamics are important to segregation process, are not shown in this image. In B, protein nodes with formins and myosins on the cortex of *S. pombe* form a dynamic actin contractile ring *in silico*. *In vivo* this ring will ingress and divide this cell. Realistic biophysical behavior for the actin filaments in this simulation may be important. For example, it is energetically favorable, because of the potential energy stored in the bending of filaments, for filaments to align themselves with the long axis of the cell. A finely tessellated and properly tuned *in silico* filament will accurately reproduce this alignment bias.

In computational experiments (in progress and to be presented elsewhere) I use this technique to model ParM and actin filaments in simple biological examples of two important cell processes, DNA segregation and cell division (Fig. 6). ParM filaments can segregate bacterial plasmids [14], [15]. This segregation occurs in cells whose longest dimension (∼3 µm) is far shorter than the ParM filament's persistence length. But filament flexibility is nevertheless important in determining the filament bundling and buckling behaviors in that model, ultimately effecting plasmid segregation competence in the model and the conclusions we reach from our simulations. Another application in progress is a detailed *in silico* study of the actin-based contraction of protein nodes in *S. pombe* –appropriately flexible actin filaments are likely critical to modeling both contractile ring formation and ingression.

As a tool for understanding cellular behavior, nano-scale agent-based modeling is in its infancy, but holds great promise. Both our factual knowledge of biological detail, and our ability to closely mimic those details *in silico* (i.e. processing power) are growing rapidly. The success of this modeling approach will depend on computationally efficient and biophysically realistic methods for representing the very many types of agents that may be present –and interacting– in a complex cellular process. The PAIRS method for *in silico* cytoskeletal filaments is one such method.

The few detailed force-based models of cellular phenomena published to date are custom-made for each biological study (though perhaps through relatively slight modification of an existing model), which is costly. At present there is no general *in silico* cellular arena sufficiently adaptable in scale, agents, and agent interactions to serve all the needs of the biological community. A development and standardization of best methods for representing each type of agent will hopefully allow for a consensus modeling framework. With such a tool biologists might routinely construct force-based nano-scale models using such an arena to test their intuition and explore the mechanisms involved in micro-scale emergent behaviors.

## Supporting Information

Text S1 Biophysical Properties of Cytoskeletal Filaments. Discusses the biophysical properties of cytoskeletal filaments that are relevant to the in silico modeling
(0.08 MB DOC)

Text S2 Rationale for using the forward Euler method. Defends use of the forward Euler method for this simulation
(0.03 MB DOC)

Text S3 Psuedo-code implementing the Pairwise Agent Interactions with Rational Superposition (PAIRS) method with tuning coefficients. Method psuedo-code
(0.05 MB DOC)

Figure S1 Deflection of beams. The expressions for expected deflection, from engineering beam theory, for simply-supported and cantilevered beams subjected to a single force applied at beam center and free end, respectively. These formulas are used to tune *in silico* biological filaments to the correct deflection under force
(0.06 MB TIF)
