# Supplementary material for: Biophysically Realistic Filament Bending Dynamics in Agent-Based Biological Simulation
Source: PLoS One. 2009 Mar 13;4(3):e4748. doi: 10.1371/journal.pone.0004748 (PMC2654463; doi:10.1371/journal.pone.0004748)
Supplement: Text S1 — Biophysical Properties of Cytoskeletal Filaments. Discusses the biophysical properties of cytoskeletal filaments that are relevant to the in silico modeling (0.08 MB DOC) [file pone.0004748.s001.doc]

## Text S1: The Biophysical Properties of Filaments

A single experimentally observable parameter, persistence length, sufficiently characterizes the filament behaviors that are most important in molecular mechanics models of cytoskeletal filaments: static deflection, bending relaxation time-constant, and thermal writhing. The theoretical expectations for static deflection and relaxation time-constant depend on bending rigidity, which is simply proportional to the persistence length. The Principle of Equipartition of Energy relates persistence length, $L_{p}$, and bending rigidity *EI* (written here as the product of elastic modulus *E* and second area moment of inertia *I*) by $k_{B}{TL}_{p}=\text{EI}$, where $k_{B}$ and *T* are Boltzmann’s constant and the absolute temperature, respectively.

*Static deflection*

Engineering beam theory [1] defines the expected deflection of a beam with bending rigidity *EI* under different loading and support conditions. Two simple cases appear to be most important for point loads experienced by filaments in the cell: simply-supported (no vertical end displacements of a horizontal beam with one end-pinned and the other free to translate horizontally, with the slopes at both ends unconstrained) and cantilevered beams (no displacement or slope at a fixed end with the other end free). Schematics of these two loading cases, as well as expressions for the expected deflection in terms of the bending rigidity *EI*, are given in Fig. S1. A section of a biological filament spanning two fixed locations might behave like a simply-supported beam (though additional slope constraints could be present), whereas a filament emanating from a network or nucleating center (e.g. an actin filament extending from a dendritic network, or a microtubule extending from a centrosome) would be best modeled as a cantilevered beam.

*Relaxation time-constant*

A dynamic system out of equilibrium, such as a cytoskeletal filament deformed from its relaxed state, will move toward that equilibrium on a certain timescale once constraining forces are removed. The time-constant characterizing this relaxation is best defined for our purposes as the time in which a system strained by measure $d$ relaxes to measure $d/e$. For a beam in a viscous fluid, and with small angle approximations, the motion is described by the hydrodynamic beam equation [2]. From the analytical solution to this equation we gain an expression for the system time-constant under different boundary conditions. For a free beam of length *L* and radius *r*, the time-constants for principal modes of bending $n=1,2,3...$ (Fig. 4A) are given by

where (1)

where is the transverse viscous drag per unit length for the filament of radius *r* a distance *h* from a plane surface, and $h$ is the viscosity [2]. Likewise, for a cantilevered filament, we expect time-constants on the order of

where (2)

*Thermal writhing*

A flexible filament immersed in a fluid will writhe as random thermal forces impinge, adopting a curved shape at any given instant. Before its bending rigidity straightens it, new thermal forces will cause it to bend into a new randomly curved shape. Thus, the filament will never come to rest. This writhing is experimentally described for biological filaments such as actin filaments and microtubules [3]. In addition, the entire filament is subject to diffusional translation and rotation. A measure of the degree of these thermally induced bending deformations is the filament’s “persistence length” --the arc length over which the time-averaged cosine of the angle between path tangents is $1/e$. Precisely, for a filament with persistence length $L_{p}$, the time average angular correlation between path tangent vectors is

(3)

where $\tilde{u}_{0}$ and $\tilde{u}_{s}$ are path tangent vectors at points separated by arc length $s$, the dot denotes a vector inner product, and the brackets denote a long time-average.

A molecular mechanics model of a filament would waste too much computer time to simulate explicitly the bombardment by an enormous number of water molecules; we instead encapsulate the ensuing deformation and bulk movement with a few randomly directed forces and torques. For a rigid body, and in the direction of any of the three principle geometric axes along which its diffusivity is $D_{\alpha}$, the mean displacement is zero and the root-mean-square displacement during time interval is $\sqrt{2D_{\alpha}Dt}$. That is, its random walk displacements for a sequence of steps of duration , come from a Gaussian distribution with zero mean and variance $2D_{\alpha}Dt$. Unless the object is a sphere or cube or similar shape with simple symmetries, the diffusivity $D_{\alpha}$ is generally different for each principle axis. The case is even more complicated for a segmented body, such as our *in silico* representation of cytoskeletal filament, but for filaments much shorter than the persistence length $L_{p}$, such as each of the rigid segments in the model of Fig. 2, the bulk Brownian movement can be well approximated by that of a rigid body of the same shape.

## References

1. Shigley JE, Mischke CR, Budynas R (2004) Mechanical Engineering Design. New York, McGraw-Hill. 1030p.

2. Howard J (2001) Mechanics of Motor Proteins and the Cytoskeleton. Massachusetts, Sinauer. 370p.

3. Gittes F, Mickey B, Nettleton J, Howard J (1993) Flexural rigidity of microtubules and actin filaments measured from thermal fluctuations in shape. J Cell Biol 120: 923-34.
