# Supplementary material for: Biophysically Realistic Filament Bending Dynamics in Agent-Based Biological Simulation
Source: PLoS One. 2009 Mar 13;4(3):e4748. doi: 10.1371/journal.pone.0004748 (PMC2654463; doi:10.1371/journal.pone.0004748)
Supplement: Text S2 — Rationale for using the forward Euler method. Defends use of the forward Euler method for this simulation (0.03 MB DOC) [file pone.0004748.s002.doc]

## Text S2: Why use the forward Euler method?

The PAIRS solution for *in silico* flexible filaments employs special elastic elements to confront a common problem in mechanistic computer simulations: the numerical instability caused by stiffness inherent in Hookean elastic elements.

*Numerical instability*

Simple Hookean springs, in which force is linearly proportional to strain, are notorious for their numerical instability (stiffness), which can make algorithms for integrating differential equations (e.g.. in the forward Euler method) uselessly slow. With too large a time-step or spring constant, the spring force overshoots its unstrained position (centers coincident) such that the distance between the bodies is greater at the end of each time-step than it was at the beginning. If unchecked, this amplification of strain will quickly cause a computer simulation to fail as some states adopt non-numeric (infinite) values. The use of sufficiently small time-step will suppress this instability, but the accompanying increase in computer time (the many more time-steps required to achieve a desired simulation time) will often be prohibitive.

The forward Euler method is the simplest of all numerical integration schemes, and the one most seriously afflicted by stiffness. More sophisticated methods can be used to cope with this type of stiffness, but all numerical schemes depend on an estimation of the local state gradient so that states can be advanced from time $t$ to $t+Dt$, assuming some rate of change. For example, to advance a state variable x through a single time-step, we need an estimate of the local velocity $v$, so that $x(t+Dt)=x(t)+vDt$. The forward Euler method just takes $v=v(t)$, whereas more advanced explicit methods calculate some average of past and present velocities, i.e. . But in molecular mechanics simulations a primary determinant of state gradients is stochastic thermal agitation, and the thermal forces a cellular body experiences at consecutive time-points are completely uncorrelated with one another. It therefore makes little sense for a simulation to include the influence of these forces at time-steps other than the present one. This is a subtle point: if inertia were not negligible, the velocities would be continuous functions of time and the positions would be differentiable functions of time. Standard ordinary differential equation (ODE) integration schemes could handle this situation. In our applications, however, where inertia is negligible, the velocities jump discontinuously every time a random thermal force impinges (i.e. every time step) and so the trajectories have non-differentiable kinks at every time step and, strictly speaking, standard ODE integration schemes fail.

To conclude, in order to avoid using extrapolations from past or future time points (which would make no sense when velocities change discontinuously), I use the forward Euler first-order method and invent special springs to deal with the issues of stiffness and discontinuous velocities.
