# Supplementary material for: Biophysically Realistic Filament Bending Dynamics in Agent-Based Biological Simulation
Source: PLoS One. 2009 Mar 13;4(3):e4748. doi: 10.1371/journal.pone.0004748 (PMC2654463; doi:10.1371/journal.pone.0004748)
Supplement: Text S3 — Psuedo-code implementing the Pairwise Agent Interactions with Rational Superposition (PAIRS) method with tuning coefficients. Method psuedo-code (0.05 MB DOC) [file pone.0004748.s003.doc]

## Text S3: Psuedo-code implementing the Pairwise Agent Interactions with Rational Superposition (PAIRS) method with tuning coefficients.

The following two pseudo-code methods demonstrate the essential aspects of implementing PAIRS. Each method would be called once for each pair of joined rigid segments. The operator signifies that the right-hand value is added to the left-hand value.

addLinkForce () {

// find the move coefficients

// find the force by PAIRS method

but

}

addLinkTorque () {

// find the torque by PAIRS method

}

applyBrownianForces () {

// appropriate random force for the rigid segment

// appropriate random torque for the rigid segment

if (segment is at end of the filament) { }

}
