# Supplementary figures and images for: Biophysically Realistic Filament Bending Dynamics in Agent-Based Biological Simulation
Source: PLoS One. 2009 Mar 13;4(3):e4748. doi: 10.1371/journal.pone.0004748 (PMC2654463; doi:10.1371/journal.pone.0004748)

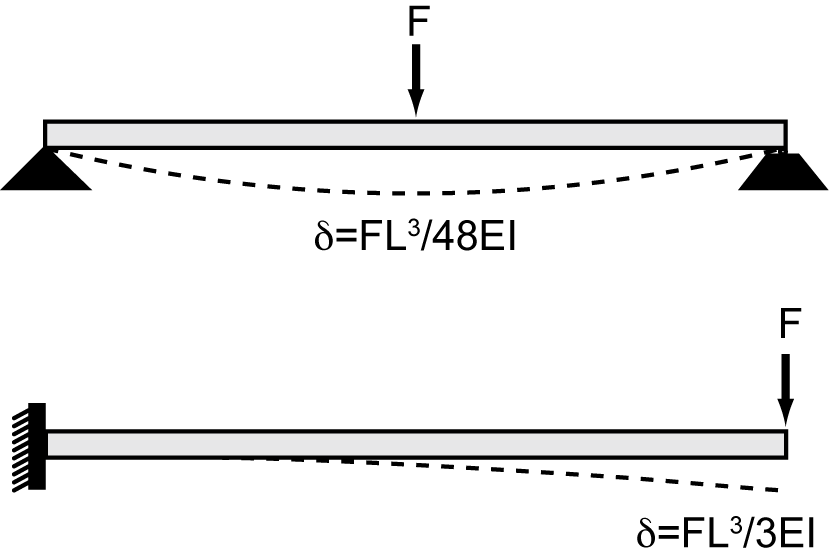

Supplement: Figure S1 — Deflection of beams. The expressions for expected deflection, from engineering beam theory, for simply-supported and cantilevered beams subjected to a single force applied at beam center and free end, respectively. These formulas are used to tune in silico biological filaments to the correct deflection under force (0.06 MB TIF) [file pone.0004748.s004.tif]
